# Supplementary figures and images for: Induced cardiomyocyte maturation: Cardiac transcription factors are necessary but not sufficient
Source: PLoS One. 2019 Oct 17;14(10):e0223842. doi: 10.1371/journal.pone.0223842 (PMC6797484; doi:10.1371/journal.pone.0223842)

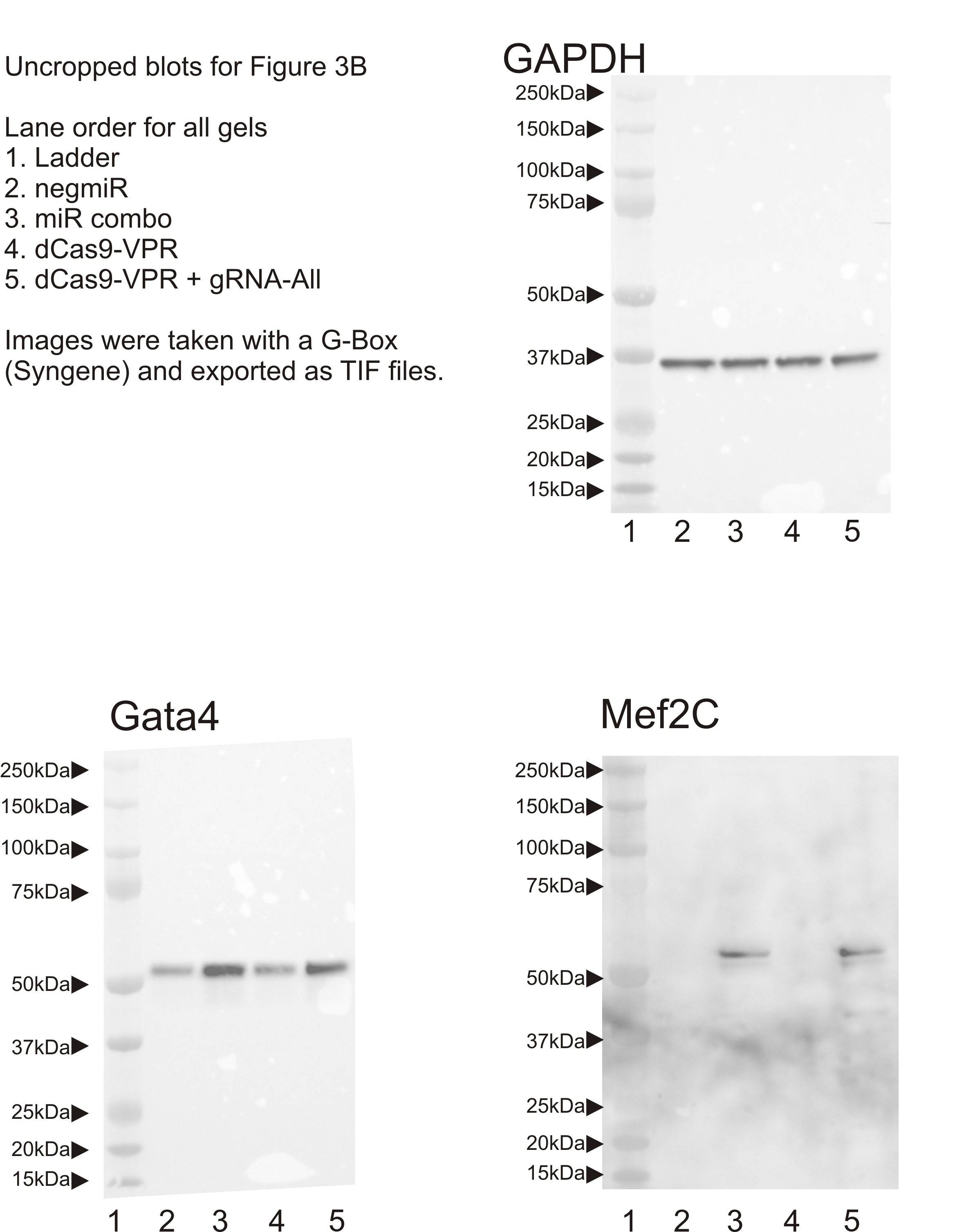

Supplement: S1 Fig — (TIF) [file pone.0223842.s001.tif]
